# Multiple Nonsyndromic Unerupted Supernumerary Teeth: A Report of a Rare Case

**DOI:** 10.1155/2022/4063856

**Published:** 2022-03-29

**Authors:** Mehrnaz Moradinejad, Alireza Hashemi Ashtiani, Vahid Rakhshan

**Affiliations:** ^1^Department of Orthodontics, Dental School, Ahvaz Jundishapur University of Medical Sciences, Ahvaz, Iran; ^2^Department of Prosthodontics, Dental School, Ahvaz Jundishapur University of Medical Sciences, Ahvaz, Iran; ^3^Department of Anatomy, Dental School, Azad University of Medical Sciences, Tehran, Iran

## Abstract

**Introduction:**

The prevalence of nonsyndromic multiple supernumerary teeth is less than 1% of all hyperdontia cases which themselves have a rather small prevalence. Cases with 10 impacted nonsyndromic supernumerary teeth are extremely rare. This report presents such a case of nonsyndromic multiple impacted supernumerary teeth.

**Case:**

A 17-year-old boy with a completely orthodontic chief complaint attended our center. He had no systemic complaints and no signs or symptoms. Oral examination showed no abnormalities. On routine pretreatment panoramic radiography, numerous impacted supernumerary teeth appeared. A CBCT showed 10 impacted vertically aligned supplementary supernumerary teeth with incomplete roots: In the maxilla, the bilateral canine-premolar areas were involved, each having 2 supernumerary teeth palatal to the permanent teeth. In the mandible, the right premolar area included 2 supernumerary teeth. The left premolar-molar area contained 3 supernumerary teeth lingual to the permanent teeth. And in the right mandibular molar area, there was a distomolar tooth distal to the third molar. No bony ankyloses, root resorptions, or fusions were observed. The surrounding bone had become thinner and the mandibular alveolar canal was involved. *Interventions*. All supernumerary teeth except a mandibular distomolar were extracted carefully before beginning the orthodontic treatment. No complications were seen after the extraction, after orthodontic treatment, and 2 years after treatment.

**Conclusions:**

The possibility of completely hidden unerupted supernumerary teeth without any signs and symptoms might highlight the value of some radiographic screenings.

## 1. Introduction

Hyperdontia (also called supernumerary teeth) is a ‘number' developmental dental anomaly, referring to any excess dental or odontological structure that is not a part of the normal dentition [1–5]. It has a prevalence ranging between 0.1% and 3.9% in permanent dentition and between 0.3 and 1.8% in primary dentition [1–5]. Hyperdontia can cause numerous esthetic and functional complications such as crowding, midline diastema, root resorption, ectopic eruption, cystic lesions, displacement of the crowns of the neighboring teeth, intraoral infections, and delayed or failed eruption [1–7], whereas, in some cases, supernumerary teeth might not disrupt occlusion or other clinical parameters and will be discerned only incidentally [7].

The etiology of hyperdontia is not known, perhaps multifactorial involving both genetic and environmental factors, with the most widely accepted hypothesis being the localized hyperactivity of the dental lamina [1, 2, 4–6, 8]. The cooccurrence of hyperdontia with numerous syndromes can indicate the genetic role; it can be seen in syndromes such as Marfan, Nance Horan, amelogenesis imperfecta, Crouzon, Franceschetti, Hallerman-Streiff, Down, Noonan, Rubenstein-Taybi, Zimmermann-Laband, Fabry, Ellis-Van Creveld, Ehlers-Danlos types III and IV, Rothmund–Thomson, Robinow, Hallermann-Streiff, Goldenhar, Gardner, incontinentia pigmenti, tricho-rhino-phalangeal syndrome, cleidocranial dysplasia, anophthalmia syndrome, craniosynostosis, orofaciodigital syndrome type I, and cleft lip and palate [1, 4, 9].

Hyperdontia usually involves few teeth: about 76-86% of patients have only 1 supernumerary tooth, while about 12-23% of patients will have only 2 supernumerary teeth [6]. Patients with more than 2 supernumerary teeth are quite rare, merely seen in about 2-8% of cases, with those having 5 or more supernumerary teeth being extremely rare and seen in less than 1% of all hyperdontia cases [5–7, 9] (perhaps about 1 in up to every 100,000 individuals, taking into account the prevalence of hyperdontia itself) [1–4, 6].

Such rare cases of multiple hyperdontia are usually a feature of syndromes and may not typically happen in nonsyndromic patients [5–7, 9]. Actually, nonsyndromic cases of multiple hyperdontia are even rarer, especially when the number of supernumerary teeth is greater than 6, and especially when all of them are impacted. We incidentally observed such an extremely rare case of 10 unerupted non-syndromic supernumerary teeth in an orthodontic patient.

## 2. Case Presentation

**Figure 1:**
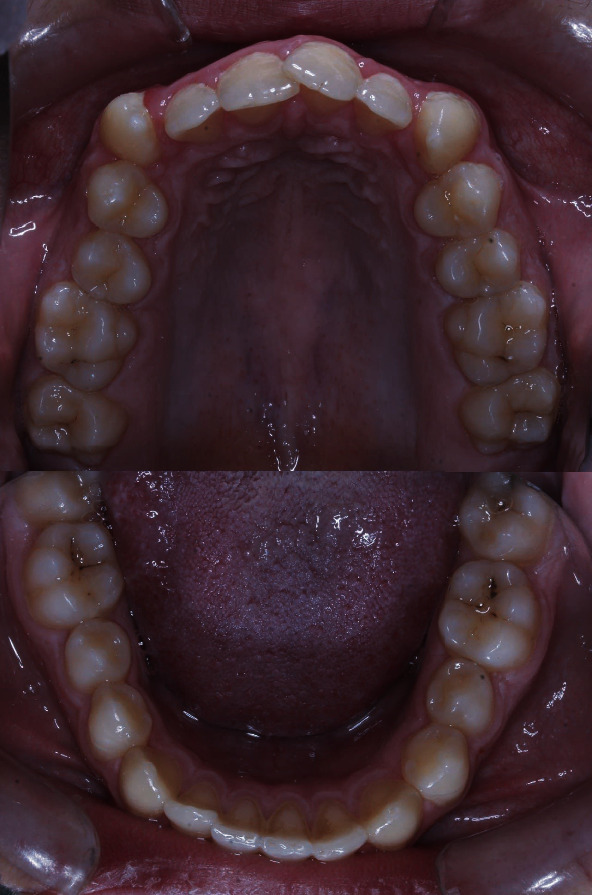
The lingual view of all teeth, showing no signs of any abnormalities.

**Figure 2:**
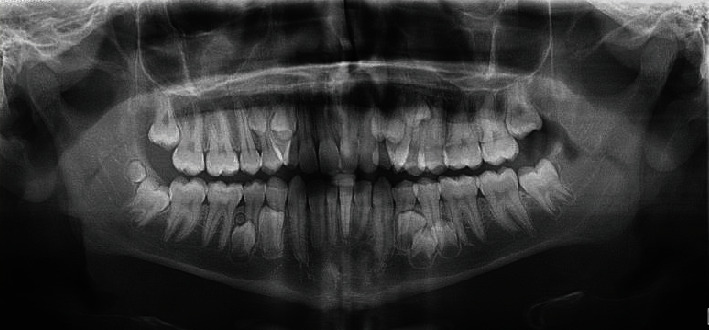
The original routine panoramic radiograph, showing supernumerary teeth.

The patient was a 17-year-old boy who had been referred to an orthodontic office. His chief complaint was that the canine teeth were protruding. His medical and family history did not indicate anything noteworthy. The person was healthy (although obese (height: 178 cm, weight: 135 kg, BMI = 42.6)) and had no complaints other than the said need for orthodontic treatment. No abnormalities were observed in his oral examination (Figure 1). No supernumerary teeth or any other anomalies were found on oral examination. For the sake of orthodontic diagnosis and treatment planning, first, a routine panoramic radiograph was prescribed. The examination of the panoramic radiograph showed evidence of supernumerary teeth (Figure 2).

**Figure 3:**
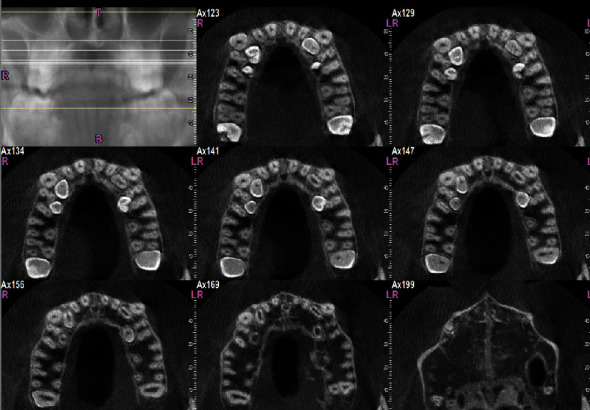
Transverse sections of the maxilla, showing sections of the maxillary supernumerary teeth.

**Figure 4:**
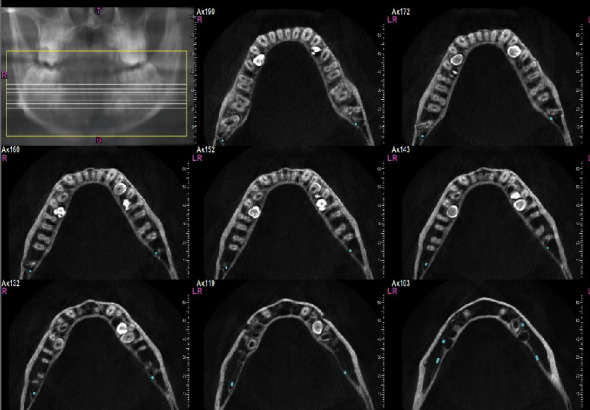
Transverse sections of the mandible, showing transverse sections of the mandibular supernumerary teeth.

**Figure 5:**
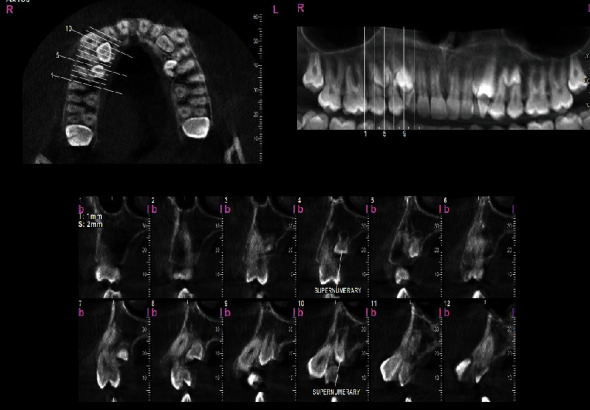
Vertical sections perpendicular to the alveolar ridge, showing the position and morphology of supernumerary teeth in the maxillary right quadrant.

**Figure 6:**
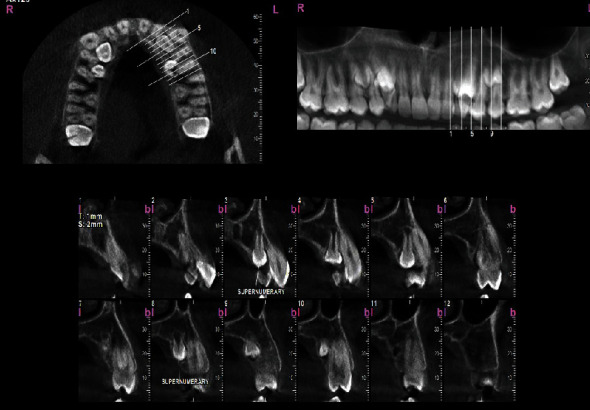
Vertical sections perpendicular to the alveolar ridge, showing the position and morphology of supernumerary teeth in the maxillary left quadrant.

**Figure 7:**
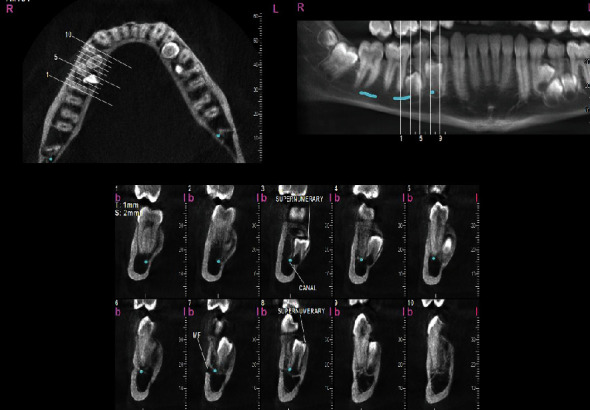
Vertical sections perpendicular to the alveolar ridge, showing the position and morphology of supernumerary teeth in the mandibular right quadrant.

**Figure 8:**
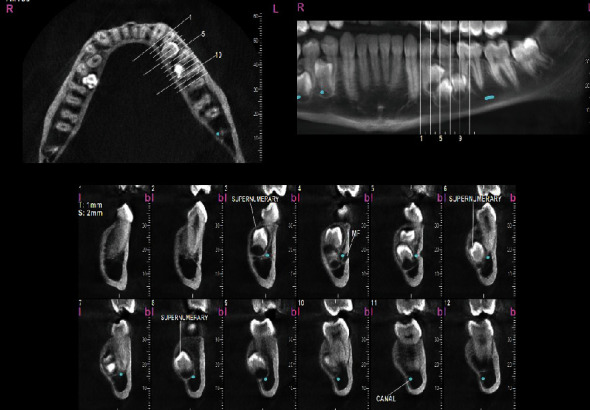
Vertical sections perpendicular to the alveolar ridge, showing the position and morphology of supernumerary teeth in the mandibular left quadrant.

**Figure 9:**
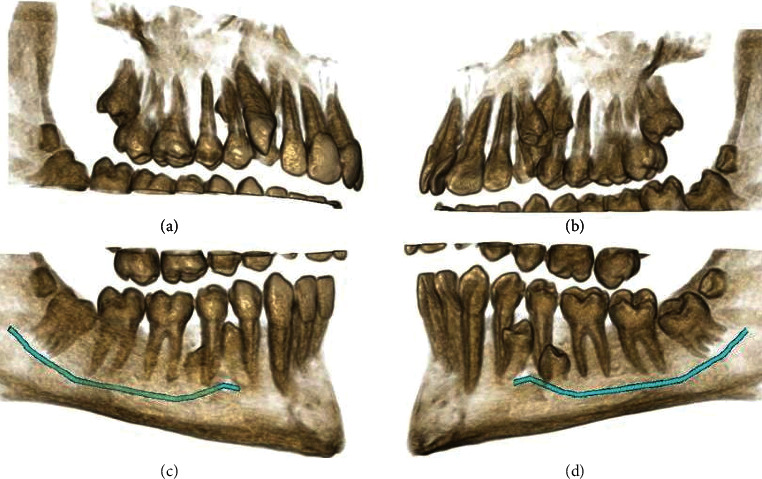
3D reconstructions of the right upper and lower quadrants. (a, c) The buccal view (with slight adjustments in the point of view). (b, d) The palatal view. The mandibular nerve canal is highlighted.

**Figure 10:**
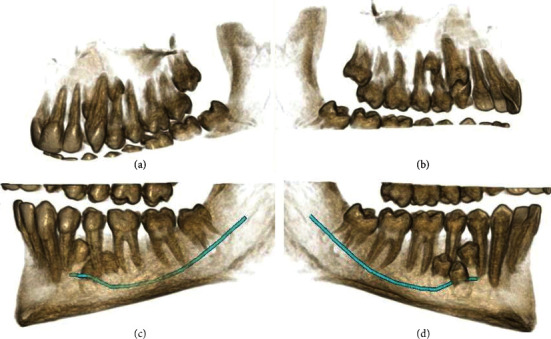
3D reconstructions of the left quadrants in the maxilla and mandible. (a, c) The buccal view. (b, d) The palatal view. The mandibular nerve canal is highlighted.

To assess the condition of the supernumerary teeth more accurately, a cone-beam computed tomography (CBCT) was taken from all bimaxillary quadrants (NewTom VGI (Quantitative Radiology, Verona, Italy), FOV = 8 × 11 cm, high resolution), which indicated the presence of 10 supernumerary teeth in different areas (Figures 3–10). The patient was also referred to a physician for the assessment of any syndromes. The physician ruled out any syndrome in the patient.

### 2.1. CBCT Assessments

**Figure 11:**
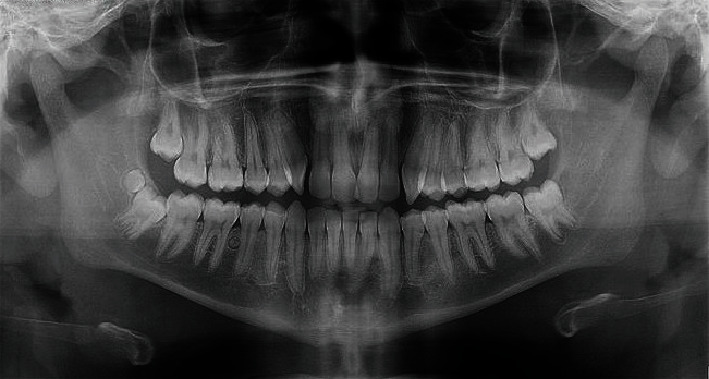
The panoramic radiograph taken after extracting 9 supernumerary teeth (all except the distomolar).

CBCT imaging was conducted. Cross-sectional, axial, coronal, and 3D images were reconstructed (Figures 4–11). The patient had all 32 normal permanent teeth. The locations of the found supernumerary teeth were as follows: In the maxilla, the bilateral canine-premolar areas were involved, each having 2 supernumerary teeth palatal to the permanent teeth. In the mandible, the right premolar area included 2 supernumerary teeth. The left premolar-molar area contained 3 supernumerary teeth lingual to the permanent teeth. And in the right mandibular molar area, there was a distomolar tooth distal to the third molar. The impaction type of all extra teeth was vertical. Anatomical features are as follows: All teeth show uncompleted roots; maxillary mesial ones had more developed roots. No bony ankylosis was detected (i.e., the periodontal ligament (PDL) space was visible around all the supernumerary teeth). Effects of supernumerary teeth on the normal teeth are as follows: No resorption and no fusion were detected at any sites. Effects of supernumerary teeth on the bone are as follows: Thinning of the palatal and lingual cortical bones were observed. Effects of supernumerary teeth on vital structures are as follows: Bilateral contacts of the roots of the supernumerary teeth with the inferior alveolar canals were observed. The supernumerary teeth had short roots, indicating that they had been developed lately. All of them were impacted, and none was erupted. All of them resembled normal teeth and hence were supplementary supernumerary teeth (there was no rudimentary hyperdontia).

### 2.2. Familial History

The patient, his parents, and his grandparents (who all were also patients attending the same dental clinic) were asked about any known cases of supernumerary teeth in any of their close or distant relatives. According to them, there was no person known for having any extra teeth among the close or distant relatives of them.

### 2.3. Orthodontic Diagnosis

The orthodontic assessments revealed that on the right side, the relationship was full-cusp molar and canine class II. On the left side, it was molar and canine class I. The lower midline was shifted to the right. Maxillary teeth were retruded.

### 2.4. Management and Any Complications

Fortunately, none of the supernumerary teeth had caused damage or root resorption to adjacent teeth, and there was no evidence of cystic or pathological changes in the extra teeth. Before starting orthodontic treatment, in order to prevent the supernumerary teeth from interfering with orthodontic movements of the teeth, all the supernumerary teeth (except the distomolar one) were extracted with the utmost care by an oral and maxillofacial surgeon under local anesthesia (Figure 11). The mandibular distomolar was not extracted because it did not interfere with orthodontic treatment or occlusion and also because its extraction might damage the surrounding tissues. After the extraction of supernumerary teeth, there were no complications on the adjacent bones and teeth.

### 2.5. Follow-Up

The orthodontic treatment was successful. No complication was seen after orthodontic treatment. The patient was followed up until two years (the present day), and he showed no problems.

## 3. Discussion

In the present case, most supernumerary teeth were located in the premolar areas. Our finding was in line with previous reports. In the case of sporadic supernumerary teeth, they usually happen in the premaxilla [2, 4, 10]. However, in the case of multiple supernumerary teeth, there might be a predominance of the hyperdontia occurrence in the premolar region (especially the mandibular premolar area), followed by the molar and the anterior areas, respectively; overall, about 60.9% of multiple supernumerary teeth may appear in the mandible, of which about 44.8% may appear in the premolar area [4, 8, 11, 12]. It is thought that supernumerary premolars might belong to a third (postpermanent) series, developing from extensions of the dental lamina [6, 13].

In this study, all the 10 supernumerary teeth were impacted without any clinical signs or symptoms and without affecting the occlusion; they were found quite accidentally. It was in line with most cases. About 75% of supernumerary premolars usually remain unerupted, with no clinical symptoms or signs, and usually discerned incidentally on radiographs [6, 7, 14–17]. Still, it should be noted that such findings have been mostly reported for cases of sporadic hyperdontia, and having 10 impacted supernumerary teeth is quite rare. All of the supernumerary teeth seen in this study were vertically positioned. In general, hyperdontia can have different directions and positions: they can stand vertically like normal teeth, appear transverse, or even inverted; they also may be ectopic or follow an abnormal eruption path [7].

Moreover, the supernumerary teeth observed in our case had short roots, indicating their late development. This finding was consistent with most supernumerary premolars which usually develop late, for instance at the age of 13 years old or later [18]. It should be noted that many supernumerary teeth are prone to recurring after extraction at a later age [6, 8]. Therefore, it is recommended to take follow-up radiographs.

The treatment may vary from author to author. The usual treatment is to conservatively extract such teeth, or if possible, to reposition some of them into the arch [5, 18]. Some suggest that if the supernumerary tooth is erupted in a reasonable alignment and has not affected the occlusion, it might be left unextracted and merely monitor until the supernumerary tooth begins to pose a danger to the neighboring structures. However, if the space is lacking, it should be extracted as early as possible to prevent crowding and occlusal disruptions [5, 19]. For impacted supernumerary teeth, an early extraction before potential orthodontic treatments may be recommended [6, 14]. Nevertheless, some experts suggest that in order to avoid any iatrogenic damage to adjacent teeth or anatomical structures, the practitioner should wait for the supernumerary tooth roots to complete and/or until the normal permanent teeth are fully erupted [5, 6, 20]. In the current case, all the supernumerary teeth except the distomolar were removed before beginning the orthodontic treatment. The distomolar was not extracted because of clinical considerations, i.e., not interfering with orthodontic treatment or occlusion and, at the same time, being traumatic to extract.

## 4. Conclusions

The possibility of occurrence of multiple unerupted supernumerary teeth without any clinical manifestations and completely hidden to the eye might emphasize the value of preventive radiography.

## Data Availability

The data are completely presented as the case report.
